# Supplementary material for: Risk assessment at work and prevention strategies on COVID-19 in Italy
Source: PLoS One. 2021 Mar 19;16(3):e0248874. doi: 10.1371/journal.pone.0248874 (PMC7978285; doi:10.1371/journal.pone.0248874)
Supplement: S1 Appendix — (DOCX) [file pone.0248874.s001.docx]

**S1 Appendix**

**Risk assessment at work and prevention strategies on COVID-19 in Italy**

**COVID-19 workplace risk assessment**

Sergio Iavicoli^1^, Fabio Boccuni^1*^, Giuliana Buresti^1^, Diana Gagliardi^1^, Benedetta Persechino^1^, Antonio Valenti^1^, Bruna Maria Rondinone^1^

^1^Department of Occupational and Environmental Medicine, Epidemiology and Hygiene, INAIL, via Fontana Candida 1, Monte Porzio Catone (Rome), Italy

*Corresponding author:

f.boccuni@inail.it (FB)

S1 Table. Occupant load factors related to the business activities and the aggregation index

| **Business activity** | **Sector (main)** | **Occupant load factors* pers/m2** | **Aggregation index** |
| --- | --- | --- | --- |
| Audience standing spaces (gyms, sporting centers, concerts) | R | 1.50 | 1.50 |
| Cinemas, theaters and congress rooms | R | 1.20-1.50 | 1.50 |
| Exhibition areas, showrooms and other temporary assembly areas | R | 1.20 | 1.50 |
| Casinos and similar gaming areas | R | 1.00 | 1.50 |
| Metro and train stations (waiting areas) | H | 1.00 | 1.50 |
| Dance halls | R | 1.00 | 1.50 |
| Places of worship | I | 0.80 | 1.30 |
| Coffee bars | I | 0.80 | 1.30 |
| Airports (waiting areas) | H | 0.72 | 1.30 |
| Theaters (stage) | R-J | 0.71 | 1.30 |
| Gyms (exercise rooms without equipment) | R | 0.71 | 1.30 |
| Restaurants | I | 0.60-0.70 | 1.30 |
| Boarding schools | P | 0.60 | 1.30 |
| Prisons | O | 0.60 | 1.30 |
| Barracks | O | 0.60 | 1.30 |
| Convents | S | 0.60 | 1.30 |
| Hotels | I | 0.60 | 1.30 |
| Bowling | R | 0.60 | 1.30 |
| University classrooms | P | 0.60 | 1.30 |
| Airports (baggage claims) | H | 0.54 | 1.30 |
| Saunas | R | 0.50 | 1.30 |
| Schools classrooms | P | 0.45-0.54 | 1.30 |
| Waiting areas (Hospitals) | Q | 0.40 | 1.30 |
| Nursery schools (childcare) | P | 0.40 | 1.30 |
| Air traffic control towers | H | 0.37 | 1.30 |
| Vocational rooms and laboratories | P | 0.30-0.40 | 1.30 |
| Public offices | O | 0.40 | 1.30 |
| Swimming pool decks | R | 0.36 | 1.30 |
| Museum | R | 0.30 | 1.30 |
| Swimming pools | R | 0.30 | 1.30 |
| Teachers rooms | P | 0.30 | 1.30 |
| Shopping centers | G | 0.20-0.25 | 1.30 |
| Gyms (exercise rooms with equipment) | R | 0.22 | 1.30 |
| Skating rinks | R | 0.22 | 1.30 |
| Reading rooms libraries | R | 0.20 | 1.15 |
| Reading rooms archives | R | 0.20 | 1.15 |
| Sport fields | R | 0.20 | 1.15 |
| Hair salons, beauty centers, laundries, pharmacies, banks | G-K | 0.20 | 1.15 |
| Sales areas of medium and large retail businesses with sector  food or mixed | G | 0.20 | 1.15 |
| Sales areas of retail businesses without food sector | G | 0.20 | 1.15 |
| Archives stack areas | O | 0.20 | 1.15 |
| Ticket offices | H-R | 0.20 | 1.15 |
| Therapies and treatments (Hospitals) | Q | 0.20 | 1.15 |
| Offices (open space) | O | 0.12 | 1.15 |
| Hospitals wards | Q | 0.12 | 1.15 |
| Library stack areas | R | 0.11 | 1.15 |
| Kitchens | I | 0.11 | 1.15 |
| Theaters (lighting and access catwalks) | R-J | 0.11 | 1.15 |
| Industrial areas | C | 0.11 | 1.15 |
| Other commercial use areas | G | 0.11 | 1.15 |
| Food shops, clothing and shoe stores, furniture stores, opticians, flower shops, photographers | G | 0.10 | 1.00 |
| Ambulatory | Q | 0.10-0.11 | 1.00 |
| Private offices | O | 0.10 | 1.00 |
| Sales areas of wholesale businesses | G | 0.10 | 1.00 |
| Sales areas of small retail businesses with specific non-food product range | G | 0.10 | 1.00 |
| Clean rooms | Q-M | 0.08 | 1.00 |
| Inpatient treatment departments | Q | 0.08-0.09 | 1.00 |
| Data processing centers | O-M | 0.08 | 1.00 |
| Single (private) offices | O-M | 0.06 | 1.00 |
| Fabrication and manufacturing areas | C | 0.05 | 1.00 |
| Medical examination rooms | Q | 0.05 | 1.00 |
| Residential areas | Q-T | 0.05 | 1.00 |
| Agricultural buildings | A | 0.04 | 1.00 |

*Adapted from UNI 1995; NFPA, 2018

S2 Table. Occupant load and aggregation index classes

| **Occupant load class** | **Aggregation index class** |
| --- | --- |
| **pers/m^2^** |  |
| **> 0.80** | **1.50** |
| **0.20 - 0.80** | **1.30** |
| **0.10 - 0.20** | **1.15** |
| **< 0.10** | **1.00** |

S1 Fig. Iso-risk curves


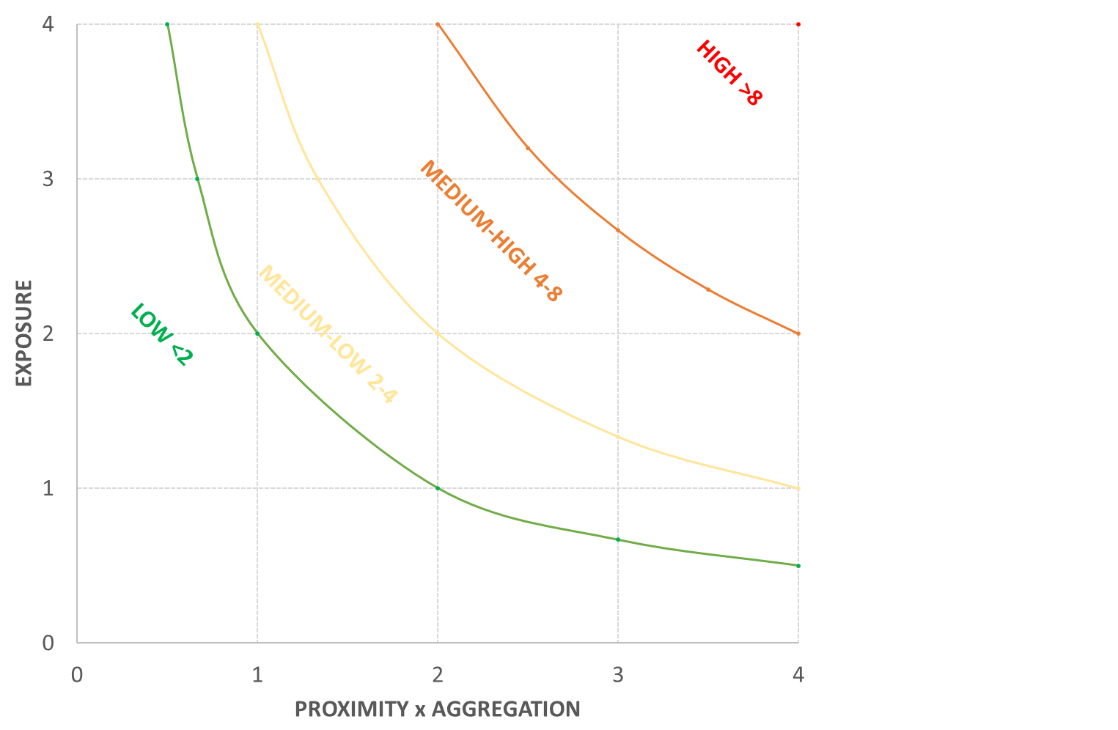


S3 Table. Summary of risk classes and aggregation index and related suspended/non suspended sectors and workers employed at 2^nd^ digit of ATECO classification.

| **ATECO 2007** | **Description** | **Aggregation index** | **Risk class** | **NON-SUSPENDED/SUSPENDED SECTORS FROM MARCH 25, 2020** | **NON-SUSPENDED/SUSPENDED SECTORS FROM APRIL 14, 2020** | **WORKERS EMPLOYEDIN NON-SUSPENDED SECTORS FROM APRIL 14, 2020 (per 1,000)** | **WORKERS EMPLOYED IN SUSPENDED SECTORS FROM APRIL 14, 2020 (per 1,000)** |
| --- | --- | --- | --- | --- | --- | --- | --- |
| **A** | **AGRICULTURE, FORESTRY AND FISHING** | | | | | | |
| 01 | CROP AND ANIMAL PRODUCTION, HUNTING AND RELATED SERVICE ACTIVITIES | 1.00 | L | NS | NS | 835 |  |
| 02 | FORESTRY AND LOGGING | 1.00 | L | S | NS | 55 |  |
| 03 | FISHING AND AQUACULTURE | 1.00 | L | NS | NS | 19 |  |
| **B** | **MINING AND QUARRYING** | | | | | | |
| 06 | EXTRACTION OF CRUDE PETROLEUM AND NATURAL GAS | 1.00 | L | NS | NS | 7 |  |
| 07 | MINING OF METAL ORES | 1.00 | L | S | S |  | 0 |
| 08 | OTHER MINING AND QUARRYING | 1.00 | L | S | S |  | 13 |
| 09 | MINING SUPPORT SERVICE ACTIVITIES | 1.00 | L | ns: 09.1 | ns: 09.1 | 3 | 2 |
| **C** | **MANUFACTURING** | | | | | | |
| 10 | MANUFACTURE OF FOOD PRODUCTS | 1.00 | L | NS | NS | 460 |  |
| 11 | MANUFACTURE OF BEVERAGES | 1.00 | L | NS | NS | 45 |  |
| 12 | MANUFACTURE OF TOBACCO PRODUCTS | 1.00 | L | S | S |  | 5 |
| 13 | MANUFACTURE OF TEXTILES | 1.00 | L | ns: 13.96.20; 13.95 | ns: 13.96.20; 13.95 | 9 | 98 |
| 14 | MANUFACTURE OF WEARING APPAREL | 1.00 | L | ns: 14.12.00 | ns: 14.12.00 | 2 | 188 |
| 15 | MANUFACTURE OF LEATHER AND RELATED PRODUCTS | 1.00 | L | S | S |  | 130 |
| 16 | MANUFACTURE OF WOOD AND OF PRODUCTS OF WOOD AND CORK, EXCEPT FURNITURE; MANUFACTURE OF ARTICLES OF STRAW AND PLAITING MATERIALS | 1.00 | L | ns: 16.24 | NS | 117 |  |
| 17 | MANUFACTURE OF PAPER AND PAPER PRODUCTS | 1.00 | L | NS (excluding codes 17.23 - 17.24) | NS (excluding codes 17.23 e 17.24) | 74 | 15 |
| 18 | PRINTING AND REPRODUCTION OF RECORDED MEDIA | 1.00 | L | NS | NS | 87 |  |
| 19 | MANUFACTURE OF COKE AND REFINED PETROLEUM PRODUCTS | 1.00 | L | NS | NS | 22 |  |
| 20 | MANUFACTURE OF CHEMICALS AND CHEMICAL PRODUCTS | 1.00 | L | NS (excluding codes: 20.12 - 20.51.01 - 20.51.02 - 20.59.50 - 20.59.60) | NS (excluding codes: 20.12 - 20.51.01 - 20.51.02 - 20.59.50 - 20.59.60) | 137 | 6 |
| 21 | MANUFACTURE OF BASIC PHARMACEUTICAL PRODUCTS AND PHARMACEUTICAL PREPARATIONS | 1.00 | L | NS | NS | 101 |  |
| 22 | MANUFACTURE OF RUBBER AND PLASTIC PRODUCTS | 1.00 | L | ns: 22.2 (excluding codes: 22.29.01 e 22.29.02) | ns: 22.2 (excluding codes: 22.29.01 e 22.29.02) | 88 | 86 |
| 23 | MANUFACTURE OF OTHER NON-METALLIC MINERAL PRODUCTS | 1.00 | L | ns: 23.13; 23.19.10 | ns: 23.13; 23.19.10 | 17 | 168 |
| 24 | MANUFACTURE OF BASIC METALS | 1.00 | L | S | S |  | 188 |
| 25 | MANUFACTURE OF FABRICATED METAL PRODUCTS, EXCEPT MACHINERY AND EQUIPMENT | 1.00 | L | ns: 25.21; 25.92 | ns: 25.21; 25.92; 25.73.01 | 72 | 505 |
| 26 | MANUFACTURE OF COMPUTER, ELECTRONIC AND OPTICAL PRODUCTS | 1.00 | L | ns: 26.6 | ns: 26.1, 26.2; 26.6 | 90 | 44 |
| 27 | MANUFACTURE OF ELECTRICAL EQUIPMEN | 1.00 | L | ns: 27.1; 27.2 | ns: 27.1; 27.2 | 68 | 132 |
| 28 | MANUFACTURE OF MACHINERY AND EQUIPMENT N.E.C. | 1.00 | L | ns: 28.29.30; 28.95.00; 28.96 | ns: 28.29.30; 28.95.00; 28.96 | 56 | 488 |
| 29 | MANUFACTURE OF MOTOR VEHICLES, TRAILERS AND SEMI-TRAILERS | 1.00 | L | S | S |  | 217 |
| 30 | MANUFACTURE OF OTHER TRANSPORT EQUIPMENT | 1.00 | L | S | S |  | 130 |
| 31 | MANUFACTURE OF FURNITURE | 1.00 | L | S | S |  | 144 |
| 32 | OTHER MANUFACTURING | 1.00 | L | ns: 32.50; 32.99.1; 32.99.4 | ns: 32.50; 32.99.1; 32.99.4 | 90 | 67 |
| 33 | REPAIR AND INSTALLATION OF MACHINERY AND EQUIPMENT | 1.15 | ML | NS (excluding codes 33.11.01; 33.11.02; 33.11.03; 33.11.04; 33.11.05; 33.11.07; 33.11.09; 33.12.92; 33.16; 33.17) | NS (excluding codes 33.11.01; 33.11.02; 33.11.03; 33.11.04; 33.11.05; 33.11.07; 33.11.09; 33.12.92) | 150 | 27 |
| **D** | **ELECTRICIT Y, GAS, STEAM AND AIR CONDITIONING SUPPLY** | | | | | | |
| 35 | ELECTRICITY, GAS, STEAM AND AIR CONDITIONING SUPPLY | 1.00 | L | NS | NS | 114 |  |
| **E** | **WATER SUPPLY, SEWERAGE, WASTE MANAGEMENT AND REMEDIATION ACTIVITIES** | | | | | | |
| 36 | WATER COLLECTION, TREATMENT AND SUPPLY | 1.00 | L | NS | NS | 38 |  |
| 37 | SEWERAGE | 1.00 | MH | NS | NS | 22 |  |
| 38 | WASTE COLLECTION, TREATMENT AND DISPOSAL ACTIVITIES; MATERIALS RECOVERY | 1.15 | ML | NS | NS | 174 |  |
| 39 | REMEDIATION ACTIVITIES AND OTHER WASTE MANAGEMENT SERVICES | 1.15 | L | NS | NS | 9 |  |
| **F** | **CONSTRUCTION** | | | | | | |
| 41 | CONSTRUCTION OF BUILDINGS | 1.00 | L | S | S |  | 420 |
| 42 | CIVIL ENGINEERING | 1.00 | L | NS (excluding 42.91; 42.99.09; 42.99.10) | NS (excluding 42.99.09; 42.99.10) | 79 | 7 |
| 43 | SPECIALISED CONSTRUCTION ACTIVITIES | 1.00 | L | ns: 43.2 | ns: 43.2 | 447 | 386 |
| **G** | **WHOLESALE AND RETAIL TRADE; REPAIR OF MOTOR VEHICLES AND MOTORCYCLES** | | | | | | |
| 45 | WHOLESALE AND RETAIL TRADE AND REPAIR OF MOTOR VEHICLES AND MOTORCYCLES | 1.15 | L | ns: 45.2; 45.3; 45.4 | ns: 45.2; 45.3; 45.4 | 321 | 96 |
| 46 | WHOLESALE TRADE, EXCEPT OF MOTOR VEHICLES AND MOTORCYCLES | 1.15 | L | ns: 46.2; 46.3; 46.46; 46.49.2; 46.61; 46.69.91; 46.69.94; 46.71 | ns: 46.2; 46.3; 46.46; 46.49.1; 46.49.2; 46.61; 46.69.91; 46.69.94; 46.71; 46.75.01 | 292 | 533 |
| 47 | RETAIL TRADE, EXCEPT OF MOTOR VEHICLES AND MOTORCYCLES | 1.15* | ML |  | ns: 47.11.1; 47.11.2; 47.11.3; 47.11.4; 47.11.5; 47.19.2; 47.2; 47.3; 47.4; 47.52.1; 47.52.2; 47.59.3; 47.61; 47.62; 47.62.1; 47.71.2; 47.73.1; 47.73.2; 47.74.0; 47.75.1; 47.76.2; 47.78.2; 47.78.4; 47.78.6; 47.91.1; 47.91.2; 47.91.3; 47.99.2 | 1,261 | 785 |
| **H** | **TRANSPORTATION AND STORAGE** | | | | | | |
| 49 | LAND TRANSPORT AND TRANSPORT VIA PIPELINES | 1.30 | ML | NS | NS | 565 |  |
| 50 | WATER TRANSPORT | 1.30** | ML | NS | NS | 41 |  |
| 51 | AIR TRANSPORT | 1.30 | H | NS | NS | 30 |  |
| 52 | WAREHOUSING AND SUPPORT ACTIVITIES FOR TRANSPORTATION | 1.15 | L | NS | NS | 313 |  |
| 53 | POSTAL AND COURIER ACTIVITIES | 1.15 | L | NS | NS | 194 |  |
| **I** | **ACCOMMODATION AND FOOD SERVICE ACTIVITIES** | | | | | | |
| 55 | ACCOMMODATION | 1.30 | L | ns: 55.1 | ns: 55.1 | 209 | 79 |
| 56 | FOOD AND BEVERAGE SERVICE ACTIVITIES | 1.30 | ML | S | S | 108 | 1,084 |
| **J** | **INFORMATION AND COMMUNICATION** | | | | | | |
| 58 | PUBLISHING ACTIVITIES | 1.15 | L | NS | NS | 62 |  |
| 59 | MOTION PICTURE, VIDEO AND TELEVISION PROGRAMME PRODUCTION, SOUND RECORDINGAND MUSIC PUBLISHING ACTIVITIES | 1.30 | L | NS | NS | 43 |  |
| 60 | PROGRAMMING AND BROADCASTING ACTIVITIES | 1.30 | L | NS | NS | 19 |  |
| 61 | TELECOMMUNICATIONS | 1.30 | L | NS | NS | 109 |  |
| 62 | COMPUTER PROGRAMMING, CONSULTANCY AND RELATED ACTIVITIES | 1.00 | L | NS | NS | 329 |  |
| 63 | INFORMATION SERVICE ACTIVITIES | 1.00 | L | NS | NS | 55 |  |
| **K** | **FINANCIAL AND INSURANCE ACTIVITIES** | | | | | | |
| 64 | FINANCIAL SERVICE ACTIVITIES, EXCEPT INSURANCE AND PENSION FUNDING | 1.00 | L | NS | NS | 388 |  |
| 65 | INSURANCE, REINSURANCE AND PENSION FUNDING, EXCEPT COMPULSORY SOCIAL SECURITY | 1.00 | L | NS | NS | 111 |  |
| 66 | ACTIVITIES AUXILIARY TO FINANCIAL SERVICES AND INSURANCE ACTIVITIES | 1.00 | L | NS | NS | 136 |  |
| **L** | **REAL ESTATE ACTIVITIES** | | | | | | |
| 68 | REAL ESTATE ACTIVITIES | 1.00 | L | S | S |  | 164 |
| **M** | **PROFESSIONAL, SCIENTIFIC AND TECHNICAL ACTIVITIES** | | | | | | |
| 69 | LEGAL AND ACCOUNTING ACTIVITIES | 1.00 | L | NS | NS | 646 |  |
| 70 | ACTIVITIES OF HEAD OFFICES; MANAGEMENT CONSULTANCY ACTIVITIES | 1.00 | L | NS | NS | 111 |  |
| 71 | ARCHITECTURAL AND ENGINEERING ACTIVITIES; TECHNICAL TESTING AND ANALYSIS | 1.00 | L | NS | NS | 398 |  |
| 72 | SCIENTIFIC RESEARCH AND DEVELOPMENT | 1.00 | L | NS | NS | 65 |  |
| 73 | ADVERTISING AND MARKET RESEARCH | 1.00 | L | S | S |  | 78 |
| 74 | OTHER PROFESSIONAL, SCIENTIFIC AND TECHNICAL ACTIVITIES | 1.00 | L | NS | NS | 196 |  |
| **N** | **ADMINISTRATIVE AND SUPPORT SERVICE ACTIVITIES** | | | | | | |
| 78 | EMPLOYMENT ACTIVITIES | 1.15 | L | ns: 78.2 | ns: 78.2 | 47 | 62 |
| 79 | TRAVEL AGENCY, TOUR OPERATOR RESERVATION SERVICE AND RELATED ACTIVITIES | 1.30 | L | S | S |  | 73 |
| 80 | SECURITY AND INVESTIGATION ACTIVITIES | 1.30 | ML | ns: 80.1; 80.2 | ns: 80.1; 80.2 | 105 | 3 |
| 81 | SERVICES TO BUILDINGS AND LANDSCAPE ACTIVITIES | 1.15 | ML | ns: 81.2 | ns: 81.2; 81.3 | 445 | 19 |
| 82 | OFFICE ADMINISTRATIVE, OFFICE SUPPORT AND OTHER BUSINESS SUPPORT ACTIVITIES | 1.15 | L | ns: 82.20; 82.92; 82.99.2; 82.99.99 | ns: 82.20; 82.92; 82.99.2; 82.99.99 | 137 | 89 |
| **O** | **PUBLIC ADMINISTRATION AND DEFENCE; COMPULSORY SOCIAL SECURITY** | | | | | | |
| 84 | PUBLIC ADMINISTRATION AND DEFENCE; COMPULSORY SOCIAL SECURITY | 1.00 | MH | NS | NS | 1,243 |  |
| **P** | **EDUCATION** | | | | | | |
| 85 | EDUCATION | 1.30 | ML | NS | NS | 1,589 |  |
| **Q** | **HUMAN HEALTH AND SOCIAL WORK ACTIVITIES** | | | | | | |
| 86 | HUMAN HEALTH ACTIVITIES | 1.30 | H | NS | NS | 1,328 |  |
| 87 | RESIDENTIAL CARE ACTIVITIES | 1.30 | MH | NS | NS | 317 |  |
| 88 | SOCIAL WORK ACTIVITIES WITHOUT ACCOMMODATION | 1.30 | H | NS | NS | 277 |  |
| **R** | **ARTS, ENTERTAINMENT AND RECREATION** | | | | | | |
| 90 | CREATIVE, ARTS AND ENTERTAINMENT ACTIVITIES | 1.50 | L | S | S |  | 93 |
| 91 | LIBRARIES, ARCHIVES, MUSEUMS AND OTHER CULTURAL ACTIVITIES | 1.30 | L | S | S |  | 51 |
| 92 | GAMBLING AND BETTING ACTIVITIES | 1.50 | MH | S | S |  | 29 |
| 93 | SPORTS ACTIVITIES AND AMUSEMENT AND RECREATION ACTIVITIES | 1.50 | ML | S | S |  | 145 |
| **S** | **OTHER SERVICE ACTIVITIES** | | | | | | |
| 94 | ACTIVITIES OF MEMBERSHIP ORGANISATIONS | 1.15 | ML | NS | NS | 178 |  |
| 95 | REPAIR OF COMPUTERS AND PERSONAL AND HOUSEHOLD GOODS | 1.15 | L | ns: 95.11.00; 95.12.01; 95.12.09; 95.22.01 | ns: 95.11.00; 95.12.01; 95.12.09; 95.22.01 | 24 | 30 |
| 96 | OTHER PERSONAL SERVICE ACTIVITIES | 1.15 | MH | S | ns: 96.01; 96.03 | 79 | 401 |
| **T** | **ACTIVITIES OF HOUSEHOLDS AS EMPLOYERS; UNDIFFERENTIATED GOODS-AND SERVICES-PRODUCING ACTIVITIES OF HOUSEHOLDS FOR OWN USE** | | | | | | |
| 97 | ACTIVITIES OF HOUSEHOLDS AS EMPLOYERS OF DOMESTIC PERSONNEL | 1.15 | MH | NS | NS | 733 |  |

* class 1.50 for shopping centers

** class 1.50 for cruise ships

NS=NON-SUSPENDED referred to whole sector;

S=SUSPENDED referred to whole sector;

ns=non-suspended referred only to a subsector

L=Low;

ML=Medium-Low;

MH=Medium-High;

H=High

S4 Table. 3^rd^ digit analysis of aggregation index and risk classes for ATECO sector G – Wholesale and retail trade; repair of moto vehicle and motorcycle

| **ATECO 2007** | **Description** | **Aggregation index** | **Risk class** |
| --- | --- | --- | --- |
| **G** | **WHOLESALE AND RETAIL TRADE; REPAIR OF MOTOR VEHICLES AND MOTORCYCLES** | 1.15 | L |
| **45** | **WHOLESALE AND RETAIL TRADE AND REPAIR OF MOTOR VEHICLES AND MOTORCYCLES** | 1.15 | L |
| **45.1** | SALE OF MOTOR VEHICLES | 1.15 | L |
| **45.2** | MAINTENANCE AND REPAIR OF MOTOR VEHICLES | 1.15 | L |
| **45.3** | SALE OF MOTOR VEHICLE PARTS AND ACCESSORIES | 1.15 | L |
| **45.4** | SALE, MAINTENANCE AND REPAIR OF MOTORCYCLES AND RELATED PARTS AND ACCESSORIES | 1.15 | L |
| **46** | **WHOLESALE TRADE, EXCEPT OF MOTOR VEHICLES AND MOTORCYCLES** | 1.15 | L |
| **46.1** | WHOLESALE ON A FEE OR CONTRACT BASIS | 1.15 | L |
| **46.2** | WHOLESALE OF AGRICULTURAL RAW MATERIALS AND LIVE ANIMALS | 1.00 | L |
| **46.3** | WHOLESALE OF FOOD, BEVERAGES AND TOBACCO | 1.00 | L |
| **46.4** | WHOLESALE OF HOUSEHOLD GOODS | 1.00 | L |
| **46.5** | WHOLESALE OF INFORMATION AND COMMUNICATION EQUIPMENT | 1.00 | L |
| **46.6** | WHOLESALE OF OTHER MACHINERY, EQUIPMENT AND SUPPLIES | 1.00 | L |
| **46.7** | OTHER SPECIALISED WHOLESALE | 1.00 | L |
| **46.9** | NON-SPECIALISED WHOLESALE TRADE | 1.00 | L |
| **47** | **RETAIL TRADE, EXCEPT OF MOTOR VEHICLES AND MOTORCYCLES** | 1.15* | ML |
| **47.1** | RETAIL SALE IN NON-SPECIALISED STORES | 1.15* | ML |
| **47.2** | RETAIL SALE OF FOOD, BEVERAGES AND TOBACCO IN SPECIALISED STORES | 1.15* | ML |
| **47.3** | RETAIL SALE OF AUTOMOTIVE FUEL IN SPECIALISED STORES | 1.15* | ML |
| **47.4** | RETAIL SALE OF INFORMATION AND COMMUNICATION EQUIPMENT IN SPECIALISED STORES | 1.15* | ML |
| **47.5** | RETAIL SALE OF OTHER HOUSEHOLD EQUIPMENT IN SPECIALISED STORES | 1.15* | ML |
| **47.6** | RETAIL SALE OF CULTURAL AND RECREATION GOODS IN SPECIALISED STORES | 1.15* | ML |
| **47.7** | RETAIL SALE OF OTHER GOODS IN SPECIALISED STORES | 1.15* | ML** |
| **47.8** | RETAIL SALE VIA STALLS AND MARKETS | 1.30 | ML |
| **47.9** | RETAIL TRADE NOT IN STORES, STALLS OR MARKETS | 1.15* | ML |

* class 1.50 if inside shopping centers

** Pharmacies with a High risk class are exception

L=Low;

ML=Medium-Low;

MH=Medium-High;

H=High

S5 Table. Workers employed in suspended sectors by gender, age and geographical area after 14 April 2020. Values (per 1,000) and percentages

| **ATECO*** | | **Gender** | | **Age** | | **Geographical Area**** | | | **TOTAL** |
| --- | --- | --- | --- | --- | --- | --- | --- | --- | --- |
|  |  | **Males** | **Female** | **Under 50** | **Over 50** | **Area 1** | **Area 2** | **Area 3** |  |
| B | Mining and quarrying | 14,16 (0,3%) | 0,85 (0,0%) | 8,45 (0,2%) | 6,56 (0,3%) | 6,46 (0,2%) | 4,58 (0,3%) | 3,96 (0,2%) | 15,00 |
| C | Manufacturing | 1969,03 (41,4%) | 668,91 (26,0%) | 1775,57 (35,3%) | 862,43 (37,5%) | 1794,61 (45,4%) | 478,56 (27,8%) | 364,84 (22,0%) | 2638,01 |
| F | Construction | 769,09 (16,2%) | 43,93 (1,7%) | 510,90 (10,2%) | 302,12 (13,1%) | 341,47 (8,6%) | 219,20 (12,8%) | 252,38 (15,2%) | 813,05 |
| G | Wholesale and retail trade; repair of motor vehicles and motorcycles | 824,45 (17,3%) | 588,22 (22,8%) | 941,23 (18,7%) | 471,43 (20,5%) | 684,00 (17,3%) | 335,57 (19,5%) | 393,10 (23,7%) | 1412,67 |
| I | Accommodation and food service activities | 611,04 (12,8%) | 551,56 (21,4%) | 902,43 (17,9%) | 260,18 (11,3%) | 498,94 (12,6%) | 319,39 (18,6%) | 344,27 (20,8%) | 1162,60 |
| L | Real estate activities | 87,77 (1,8%) | 76,26 (3,0%) | 96,97 (1,9%) | 67,06 (2,9%) | 82,61 (2,1%) | 49,43 (2,9%) | 31,99 (1,9%) | 164,03 |
| M | Professional, scientific and technical activities | 34,86 (0,7%) | 43,25 (1,7%) | 60,21 (1,2%) | 17,89 (0,8%) | 50,78 (1,3%) | 18,02 (1,0%) | 9,3 (0,6%) | 78,10 |
| N | Administrative and support service activities | 133,42 (2,8%) | 160,26 (6,2%) | 203,72 (4,0%) | 89,96 (3,9%) | 139,47 (3,5%) | 89,09 (5,2%) | 65,12 (3,9%) | 293,68 |
| R | Arts, entertainment and recreation | 183,93 (3,9%) | 134,26 (5,2%) | 227,15 (4,5%) | 91,02 (4,0%) | 142,19 (3,6%) | 104,32 (6,1%) | 71,68 (4,3%) | 318,19 |
| S | Other service activities | 127,75 (2,7%) | 303,11 (11,8%) | 300,9 (6,0%) | 129,96 (5,6%) | 211,16 (5,3%) | 100,45 (5,8%) | 119,26 (7,2%) | 430,87 |
| T | Activities of households as employers; undifferentiated goods-and services-producing activities of households for own use | 0,72 (0,0%) | 4,85 (0,2%) | 3,32 (0,1%) | 2,26 (0,1%) | 3,20 (0,1%) | 0,55 (0,0%) | 1,82 (0,1%) | 5,57 |
|  | **Total** | **4756,22 (100,0%)** | **2575,46 (100,0%)** | **5030,85 (100,0%)** | **2300,86 (100,0%)** | **3954,89 (100,0%)** | **1719,16 (100,0%)** | **1657,72 (100,0%)** | **7331,77** |

***Non-suspended sectors:**

**A – AGRICULTURE, FORESTRY AND FISHING** 01; 02; 03

**B – MINING AND QUARRYING** 05; 06; 09.1

**C – MANUFACTURING** 10; 11; 13.96.20; 13.95; 14.12.00; 16; 17 (excluding codes 17.23; 17.24); 18; 19; 20 (excluding codes 20.12; 20.51.01; 20.51.02; 20.59.50; 20.59.60); 21; 22.2 (excluding codes 22.29.01; 22.29.02); 23.13; 23.19.10; 25.21; 25.73.1; 25.92; 26.1; 26.2; 26.6; 27.1; 27.2; 28.29.30; 28.95.00; 28.96; 32.50; 32.99.1; 32.99.4; 33 (excluding codes 33.11.01; 33.11.02; 33.11.03; 33.11.04; 33.11.05; 33.11.07; 33.11.09; 33.12.92)

**D – ELECTRICITY, GAS, STEAM AND AIR CONDITIONING SUPPLY** 35

**E – WATER SUPPLY, SEWERAGE, WASTE MANAGEMENT AND REMEDIATION ACTIVITIES** 36; 27; 38; 39

**F – CONSTRUCTION** 42 (excluding codes 42.99.09; 42.99.10); 43.2

**G – WHOLESALE AND RETAIL TRADE; REPAIR OF MOTOR VEHICLES AND MOTORCYCLES** 45.2; 45.3; 45.4; 46.2; 46.3; 46.46; 46.49.1; 46.49.2; 46.61; 46.69.91; 46.69.94; 46.71; 46.75.01. Non-suspended 47.11.1; 47.11.2; 47.11.3; 47.11.4; 47.11.5; 47.19.2; 47.2; 47.3; 47.4; 47.52.1; 47.52.2; 47.59.3; 47.62.1; 47.73.1; 47.73.2; 47.74.0; 47.75.1; 47.76.2; 47.78.2; 47.78.4; 47.78.6; 47.91.1; 47.91.2; 47.91.3; 47.99.2; 47.71.2; 47.61; 47.62

**H – TRANSPORTATION AND STORAGE** 49; 50; 51; 52; 53

**I – ACCOMMODATION AND FOOD SERVICE ACTIVITIES** 55.1

**J – INFORMATION AND COMMUNICATION** 58; 59; 60; 61; 62; 63

**K – FINANCIAL AND INSURANCE ACTIVITIES** 64; 65; 66

**M – PROFESSIONAL, SCIENTIFIC AND TECHNICAL ACTIVITIES** 69; 70; 71; 72; 74; 75

**N – ADMINISTRATIVE AND SUPPORT SERVICE ACTIVITIES** 78.2; 80.1; 80.2; 81.2; 81.3; 82.20; 82.92; 82.99.2; 82.99.99

**O – PUBLIC ADMINISTRATION AND DEFENCE; COMPULSORY SOCIAL SECURITY** 84

**P – EDUCATION** 85

**Q – HUMAN HEALTH AND SOCIAL WORK ACTIVITIES** 86; 87; 88

**S – OTHER SERVICE ACTIVITIES** 94; 95.11.00; 95.12.01; 95.12.09; 95.22.01Non-suspended 96.01; 96.03

**T –ACTIVITIES OF HOUSEHOLDS AS EMPLOYERS; UNDIFFERENTIATED GOODS-AND SERVICES-PRODUCING ACTIVITIES OF HOUSEHOLDS FOR OWN USE** 97

** **Area 1:** Piedmont, Lombardy, Veneto, Emilia-Romagna, Marche

**Area 2:** Aosta Valley, Trentino South Tyrol, Friuli-Venezia Giulia, Liguria, Tuscany, Umbria, Lazio

**Area 3:** Abruzzo, Molise, Campania, Apulia, Basilicata, Calabria, Sicily, Sardinia
